# Wearable Tendon Kinetics

**DOI:** 10.3390/s20174805

**Published:** 2020-08-26

**Authors:** Sara E. Harper, Rebecca A. Roembke, John D. Zunker, Darryl G. Thelen, Peter G. Adamczyk

**Affiliations:** 1 Department of Biomedical Engineering, University of Wisconsin—Madison, Madison, WI 53706, USA; seharper@wisc.edu (S.E.H.); dgthelen@wisc.edu (D.G.T.); 2 Department of Mechanical Engineering, University of Wisconsin—Madison, Madison, WI 53706, USA; rroembke@wisc.edu (R.A.R.); jzunker@wisc.edu (J.D.Z.)

**Keywords:** noninvasive, field-based measurement, locomotion, muscle-tendon mechanics, Achilles, shear wave tensiometry

## Abstract

This study introduces a noninvasive wearable system for investigating tendon loading patterns during outdoor locomotion on variable terrain. The system leverages shear wave tensiometry, which is a new approach for assessing tendon load by tracking wave speed within the tissue. Our wearable tensiometry system uses a battery-operated piezoelectric actuator to induce micron-scale shear waves in a tendon. A data logger monitors wave propagation by recording from two miniature accelerometers mounted on the skin above the tendon. Wave speed is determined from the wave travel time between accelerometers. The wearable system was used to record Achilles tendon wave speed at 100 Hz during 1-km outdoor walking trials in nine young adults. Inertial measurement units (IMUs) simultaneously monitored participant position, walking speed, and ground incline. An analysis of 5108 walking strides revealed the coupled biomechanical effects of terrain slope and walking speed on tendon loading. Uphill slopes increased the tendon wave speed during push-off, whereas downhill slopes increased tendon wave speeds during early stance braking. Walking speed significantly modulated peak tendon wave speed on uphill slopes but had less influence on downhill slopes. Walking speed consistently induced greater early stance wave speeds for all slopes. These observations demonstrate that wearable shear wave tensiometry holds promise for evaluating tendon tissue kinetics in natural environments and uncontrolled movements. There are numerous practical applications of wearable tensiometry spanning orthopedics, athletics, rehabilitation, and ergonomics.

## 1. Introduction

Wearables are transforming the field of biomechanics, with the capacity to measure real-world movement enabling new applications in sports [1,2], ergonomics [3,4,5], and rehabilitation [6,7,8,9]. The overwhelming majority of studies to date have used wearable inertial sensors to measure movement kinematics. These common raw measurements have been analyzed in many ways, such as quantifying sports performance [10,11], monitoring symptoms of movement disorders [12,13], analyzing gait [14,15,16] or losses of balance [17], or estimating markers of injury risk, such as tibial shock [18,19], all in non-laboratory settings. While informative, it is challenging to use kinematics to infer the underlying joint and tissue loading. Muscle-tendon kinetics are of particular interest for quantifying performance, assessing injury risk, and evaluating rehabilitation progress. However, there remain no viable approaches to measure the forces transmitted by muscle-tendon units during human movement outside a laboratory.

Biomechanics laboratory instrumentation, such as motion capture, dynamometry, and electromyography (EMG) systems, remain the de facto standards for assessing kinetics. With input from these modalities, musculoskeletal models can be used to estimate the underlying muscle and tendon tissue loads. However, these methods are time consuming, require great expense and expertise to use, and produce kinetic resultsthat are limited by the assumptions and simplifications made during the processes of data collection and analysis [20,21,22,23]. Direct sensors of tendon loads, e.g., implanted tendon buckles and fiber optic cables, have been introduced, but the applicability of these technologies is limited by their invasiveness [24,25,26,27,28,29]. As a result, tendon kinetics analysis remains primarily a research tool used for a limited range of activities performed in a laboratory space.

Wearable sensors have been used to approximate traditional kinetics analysis outside the laboratory. For example, inertial measurement units (IMUs) can be used to record whole-body movement and used with a full-body model to estimate external ground reaction forces under certain circumstances [30,31]. However, these techniques are not yet proven for arbitrary tasks or body types. Alternatively, instrumented shoes and insoles have been introduced to directly record ground reactions [20,32,33,34,35], but this approach requires specific footwear that is generally bulky and may not be suited to different tasks. In all cases, external kinetics must still be processed through a biomechanical model to estimate internal tissue loads [19,36,37], and thereby are dependent on the same complex models and assumptions that limit laboratory approaches.

There is a need for noninvasive sensor technologies that can more directly assess musculotendon kinetics without musculoskeletal models and additional instrumentation. Sensors that measure sound wave transmission in tendon can provide an assessment of tissue elasticity, which exhibits a nonlinear dependence on loading [38]. However, sound waves travel at speeds around 2000 m/s in tendon, which are challenging to measure with skin-mounted sensors. High frame rate ultrasonic imaging can be used to measure shear wave speed in tissues [39], which are an order of magnitude slower than sound wave speed. Ultrasonic measurements of shear wave speed have been shown to increase monotonically with load in both muscle [40] and tendon [41]. However, shear wave ultrasound equipment tends to be bulky and have limited sample rates that are not suitable for dynamic movement. We recently introduced a skin-mounted accelerometer-based sensor that estimates tendon loading by tracking wave propagation in the tendon tissue under the skin [42,43]. The sensor is composed of an actuator that induces waves in subcutaneous tendons, and miniature accelerometers that track wave propagation speed [42]. Wave speed varies monotonically with tendon tension, similarly to how the vibration frequency of a guitar string changes with tautness. This shear wave tensiometer (SWT) enables estimation of tendon tissue loading without the complex models and analyses required with traditional instruments. We used SWT to measure variations in Achilles, patellar, and hamstring tendon tension in treadmill walking and running [42] with no need for other instrumentation.

Adapting tendon tensiometry to a wearable form would enable such measurements in much more varied and interesting scenarios. Real-world movement involves activities and terrains that cannot practically be reproduced in a laboratory but are as important as steady-state locomotion to everyday mobility and performance. To address this need, one recent effort introduced a wearable system that estimates tendon tension using waveform analysis of short bursts of tendon vibrations [44]. However, the system has a low sample rate (5 Hz) and requires extensive training data inside a laboratory, reducing its applicability for tracking transient or unique movements. Extending the high-rate calibration-free SWT approach to a wearable device would bring the scientific and practical benefits of tendon load tracking to the study of dynamic movements in arbitrary settings and activities. Such technology would enable many applications beyond basic biomechanical science, including health care, sports performance, and ergonomics.

The purpose of this study was to introduce a wearable version of the shear wave tensiometry system with a high sample rate and sufficient dynamic range for tracking unconstrained locomotion. We used this system to investigate continuous stride-by-stride Achilles tendon loading patterns during outdoor walking on terrain of varying ground incline. We aimed to determine whether the wearable can detect incline- and speed-related changes in Achilles loading that match trends in ankle moment measured in previous lab-based studies. To do so, we analyzed the systematic effects of ground incline and walking speed on Achilles wave speed patterns. We hypothesized that wearable tensiometry will measure increasing peak tendon wave speed as the speed increases and as the slope increases from downhill to uphill. We explore analytical approaches newly enabled by coupling large data sets of tendon kinetics and real-world terrain characteristics. Finally, we discuss the strengths and limitations of this approach and describe several potential applications.

## 2. Materials and Methods

**Figure 1:**
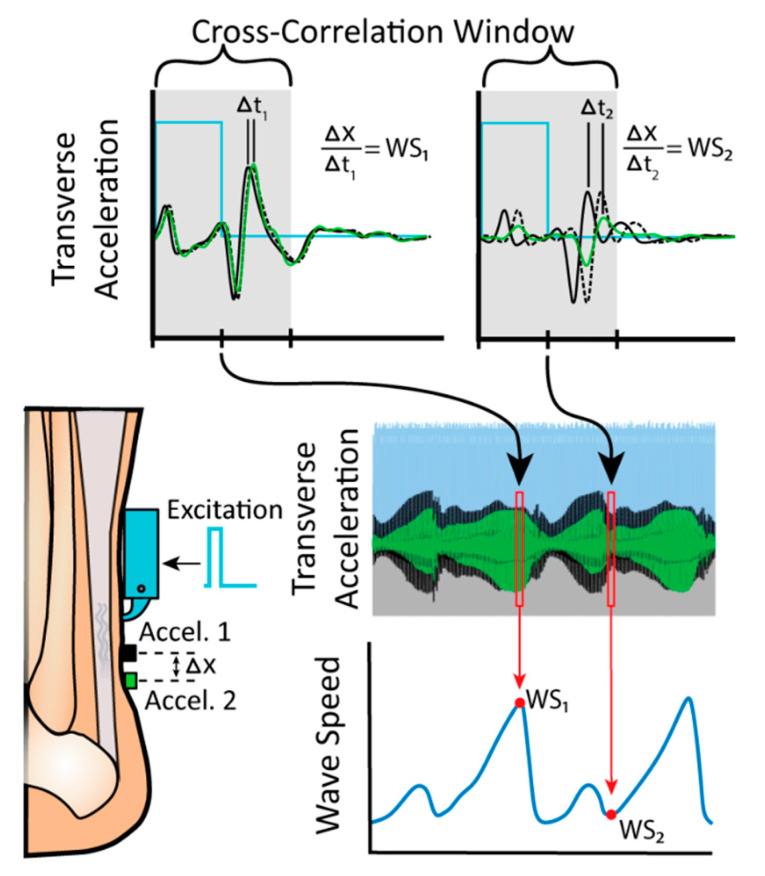
The tapper mechanism was driven by a 100 Hz pulse wave (2 ms pulse, the time required for one wave to dissipate) excitation signal. The impulsive tap induces a shear wave in the Achilles tendon that is recorded as it passes two distal accelerometers mounted 8 mm (Δ*x*) apart. Cross correlation of the accelerometer signals in a 4-ms window after the excitation onset was used to compute the wave travel time (Δ*t*), which in turn was used to compute the wave speed (WS).

The wearable shear wave tensiometry system consisted of a piezo driver box, a piezoelectric tapping mechanism, an accelerometer array, and a data logger (Figure 1). A custom battery-powered circuit used a micro piezo driver (PDu100, PiezoDrive, Shortland, NSW, Australia) to generate a 100 Hz, 20% duty cycle pulse wave. The pulse wave excitation signal drove an internal piezoelectric actuator stack (PK4JQP2, Thorlabs, Newton, NJ, USA) embedded in a tendon-tapping mechanism consisting of a tap head mounted on a rotational pin joint, with a 2:1 movement amplification lever. The accelerometer array consisted of two miniature accelerometers (Model 352C23, PCB Piezotronics, Depew, NY, USA) embedded in small plastic housings to contact the skin with their sensing axes normal to the skin surface and spaced 8 mm apart in a silicone mold (Mold Star 15 SLOW, Smooth-On, Macungie, PA, USA). The tap signal and accelerometry data were collected at 51.2 kHz via a battery-powered data logger (WebDAQ 504, Measurement Computing Corporation, Norton, MA, USA). Accelerometer signals were bandpass filtered (150 to 1500 Hz) using a fourth-order zero-lag Butterworth filter. Cross-correlation analysis of the accelerometer signals for 4 ms after each tap was used to ascertain the wave travel time between the two accelerometers. Shear wave speed was calculated as the quotient of the distance and wave travel time, as shown in Figure 1. Wave speeds in our study varied from roughly 15 to 100 m/s, which translates to a wave propagation delay of 28 to 4 samples, respectively. Cosine fitting of the cross-correlation permits subsample resolution in determining optimal signal delay, resulting in theoretical errors less than 1% of the time delay [45].

**Figure 2:**
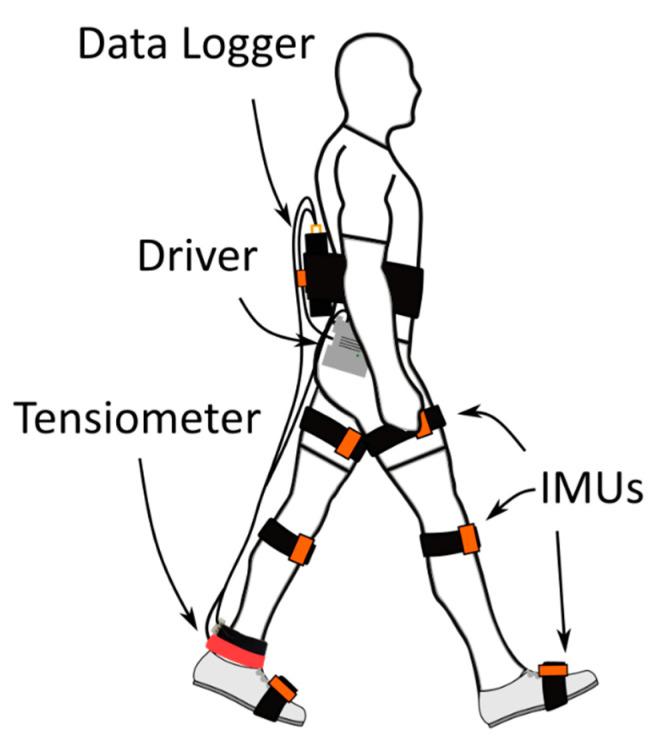
Portable instrumentation used in the outdoor walking tests. A shear wave tensiometer was mounted over the right Achilles tendon, wired to a battery-powered piezo driver and data logger mounted on the waist and low back. Stride length, walking speed, and position along the prescribed walking course were recorded from the XSens lower-body IMU model (XSens MVN Analyze software; IMUs shown in orange).

Twelve healthy young adults participated in the study after giving their written informed consent. The study was conducted in accordance with the Declaration of Helsinki, and the protocol was approved by the University of Wisconsin-Madison Institutional Review Board (protocol 2018-0487, approved 15 October 2019). Of these, nine yielded complete data sets (3F/6M, 23.4 ± 1.9 years, height 1.8 ± 0.12 m, body mass 76.4 ± 11.3 kg) and three were discarded due to data loss or shifting of the instrument, suspected to be caused by inadequate contact between the tensiometer and tendon. The tapping mechanism and accelerometer were positioned over the right Achilles tendon and secured via neoprene straps and self-adherent wrap, respectively. Inertial measurement units (MVN Awinda, XSens B.V., The Netherlands) were attached bilaterally to the feet, shanks, and thigh segments. The piezo driver, data logger, battery, and an additional inertial measurement unit were all mounted on a waist pack, as shown in Figure 2.

**Figure 3:**
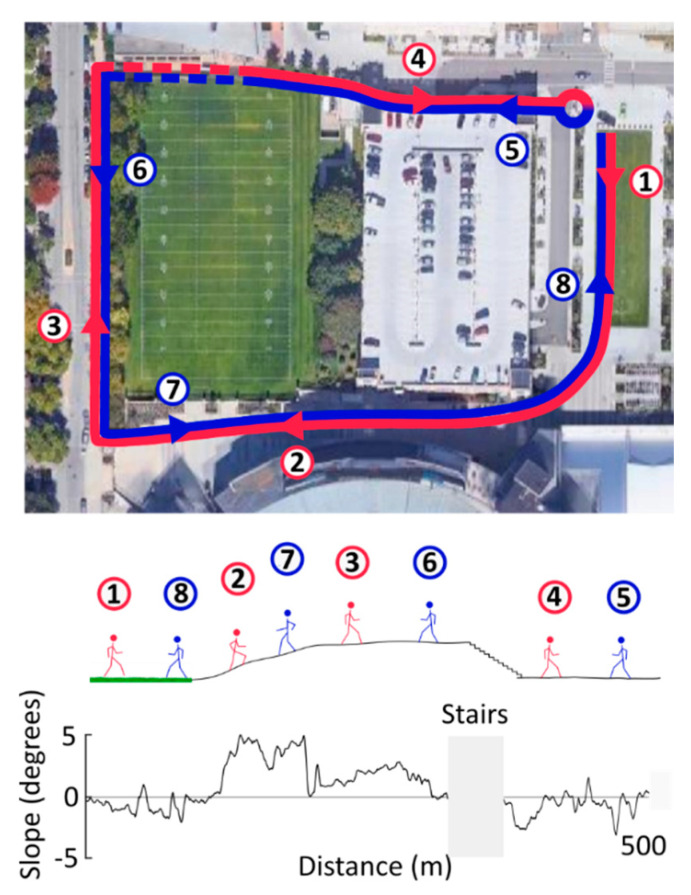
Aerial view of the 1-km outdoor walking course, including ground inclines, declines, and stairs. Four of the participants started near the edge of the grass and walked a lap according to the figure shown. They then turned around and walked the same course in the opposite direction. The remaining five participants walked the course in the opposite direction (5 to 8, then 1 to 4). The ground incline data when traversing the course in clockwise direction is shown in the bottom trace. Stairs, shown in broken lines (top) or hidden (bottom), were excluded from the analysis due to faulty data and behavioral variability.

Participants first acclimated to the system while walking on a treadmill. Then, they went outdoors to perform a walking test on a predetermined 1-km outdoor course that included grass, level sidewalk, and paved inclines between −5° and 5°, as shown in Figure 3. Participants walked this loop at a comfortable speed in both clockwise and counterclockwise directions, with the starting direction randomized for each participant. Stair segments were excluded from analysis due to faulty data and behavioral variability.

Achilles shear wave tensiometer data were continuously collected at 51.2 kHz during the trials together with a pulse wave time-synced to the piezo drive signal. Whole-body movement was recorded from the wearable inertial sensors and reconstructed using proprietary algorithms (MVN Analyze, XSens B.V., The Netherlands). Quantities of interest from these data were the participant position along the prescribed course, stride length, and instantaneous walking speed. Using a thresholding technique, low-pass-filtered data were used to identify stride timing. For the kinematic data, the threshold was set at 30% of the average peak value of foot velocity; for the tensiometry data, the threshold was set at 70% of the average peak value of wave speed. For both kinematic and tensiometry data, stride initiation was defined by the midpoint between successive falling and rising threshold crossings. This resulted in an equal number of strides from both sets of data, allowing for them to be paired for analytical purposes. Tendon wave speed data were then parsed into individual strides and normalized to 101 data points per stride.

To measure ground incline, we secured an IMU to the crossbar of a bicycle and recorded both position (GPS) and orientation (IMU) as the bicycle was slowly pushed over the 1-km course. The position and sensor orientation data were used to define the ground incline at every point in the course, resulting in a topographical look-up table; this was used to ascertain the ground incline for each stride of a participant based on their position along the course. To account for drift of the reconstructed path, the position data were scaled to the known distance of each course segment (separated by corners and stairs).

Ground inclines were assigned to each stride using the path reconstructed from IMU data and the topographical look-up table. Course segments were extracted from participant position data and scaled to the known distance of the corresponding segment. Using the time of each stride obtained from the wave speed thresholding technique, the stride number was used to determine the location of the participant at each stride. Finally, a ground incline value from the course was assigned to each stride, corresponding to the distance along the course. Strides were binned into ground incline categories of −4°, −2°, 0°, +2°, and +4° for averaging. These bins were defined as follows: Less than −3°, −3° to −1°, −1° to +1°, +1° to +3°, and greater than +3°. The peak tendon wave speed was measured from every stride in the zero-degree category and used to estimate the mean peak wave speed for each participant in level walking. Wave speed data for each participant were then normalized to these individual mean peak values.

These data were used to assess the sensitivity of the peak Achilles tendon wave speed to ground incline and walking speed. First, we compared peak wave speed values across different ground incline bins using repeated-measures analysis of variance (ANOVA), treating slope as a fixed factor and subject as a random factor, with pairwise post-hoc *t*-tests and a significance level set to α = 0.05. Next, we performed a linear regression of peak wave speed versus unbinned ground incline angle of each stride, to determine a numerical sensitivity (slope of the regression). We computed a regression for each individual as well as for the whole population (Appendix B), using a significance level α = 0.05 to determine whether the regression coefficient was significantly different from zero. We also performed a multiple linear regression of the peak wave speed as a function of both the unbinned ground incline angle and walking speed of each stride, including a multiplicative interaction. We used the regression to create a contour plot to illustrate the combined effects of speed and slope on peak wave speed. We repeated the multiple linear regression for mean mid-stance wave speed (mean from 10% to 30% stride) and again created a contour plot. Finally, to illuminate continuous differences in loading profiles as a function of the ground incline angle, we used statistical parametric mapping (SPM) to conduct two-sample *t*-tests comparing continuous wave speed signals from each binned incline condition against level walking, including appropriate adjustments for non-independence of sequential samples [46,47,48]. SPM computes a continuous test statistic (SPM{t}) indicating the probability that a difference between smooth random curves would be as extreme as that observed at each point. Regions of statistical difference (α < 0.05) were determined throughout the gait cycle, wherever the test statistic exceeded the critical threshold t* for 5% probability. We plotted example SPM results comparing the +4° condition to the 0° condition, and a comparison of 0° outdoor vs. level treadmill walking to assess the equivalence of these conditions.

## 3. Results and Discussion

**Figure 4:**
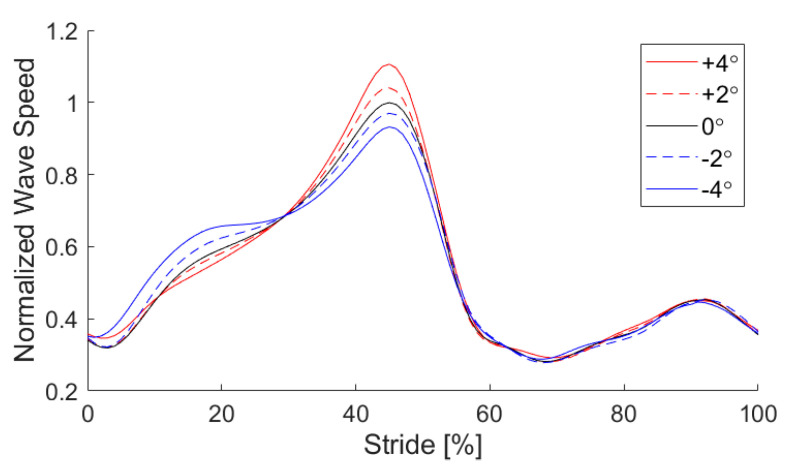
Ensembled Achilles tendon wave speed patterns at binned inclines. Normalization is to mean peak wave speed in the level walking condition for each participant. Peak wave speed of each stride was aligned to 45% of the stride to approximate a traditional gait cycle for visualization [49].

We successfully used shear wave tensiometry to track Achilles wave speed patterns during outdoor walking on variable terrain. Participants completed the course in 13.0 ± 0.9 min (mean ± standard deviation) with an average speed of 1.46 ± 0.16 m/s. From these trials, we analyzed 5108 strides across the 9 participants who participated. Excluding the section of stairs, the path was measured at 961.7 m, was traversed with a stride length of 1.55 ± 0.13 m (mean ± standard deviation, SD), and thus yielded an average of 620 strides per participant. On average, 53 strides per participant were excluded due to either faulty data or improper footfall detection from the thresholding method. Wave speed patterns and magnitudes were comparable to that observed in laboratory studies [42,49]. The average maximum and minimum wave speeds over all gait cycles were 66 and 16 m/s, respectively, with the peak wave speed aligning with the push-off, and minimal values tending to occur just after toe-off, as shown in Figure 4. We used the mean peak wave speed from strides in the 0° bin as the normalization factor for each participant; individual values are summarized in Appendix A, Table A1. Individual participants’ gait characteristics are summarized in Appendix A, Table A2.

We accomplished our goal of using tensiometry to track slope and speed-dependent variations in tendon loading patterns. As shown in Figure 4, ensemble wave speeds binned to terrain slope exhibited distinct features during midstance and push-off that correspond with biomechanical demands. During midstance (roughly 20% stride cycle), tendon wave speed magnitudes were elevated on declines and reduced on inclines. This corresponds with increased early Achilles tendon loading during this braking phase, as the ankle tries to slow the body’s downhill advance; increased Achilles loading drives the center of pressure further forward and results in a rearward ground reaction force [50]. During push-off (peak; 45% stride), wave speeds were elevated on inclines as seen in Figure 4, reflecting an increased loading required to propel the body uphill. Qualitatively, these incline-dependent variations in Achilles tendon wave speed patterns concur with biomechanical expectations and are generally consistent with lab-based metrics [50,51]. Quantitatively, we detected a 10.7 ± 12.6% (mean ± SD), 4.2 ± 10.0%, −3.0 ± 8.4%, and −6.8 ± 10.7% change in push-off peak wave speed at the 4°, 2°, −2°, and −4° conditions, respectively, relative to the 0° condition (*p* < 10^−6^). Assuming that tendon load is proportional to wave speed squared [42], this corresponds with loading changes of 22.5 ± 21.2%, 8.5 ± 18.6%, −5.8 ± 17.0%, and −13.1 ± 19.3%. Despite the relatively large variance, the mean differences are significant and meaningful because of the large number of accumulated samples. These findings are comparable to prior laboratory studies showing a 15% increase in peak ankle moment at 5° incline conditions [52].

**Figure 5:**
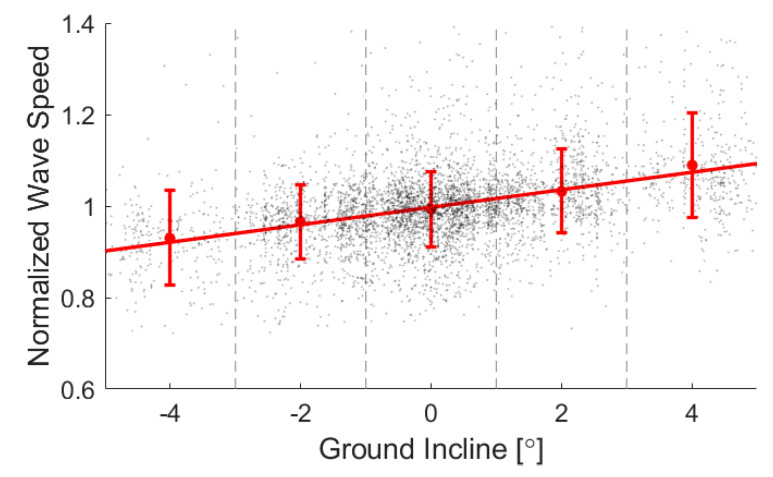
A regression of peak loading against ground incline yielded a positive association (*p* < 10^−6^). Means and standard deviations for each binned incline demonstrate the trend, while the spread of data shows the remaining variability in the data. Regression results for individual subjects are shown in Appendix B.

Real-world locomotion data allow one to examine the sensitivity of biomechanical metrics to continuously varying environmental factors. We used our stride-by-stride data to investigate the relationship between ground incline and peak wave speed, as displayed in Figure 5. Individual and group regressions are summarized in Appendix B, Table A3. This analysis revealed a highly significant sensitivity (*p* < 10^−6^) of wave speed to incline, with each 1° increment in grade associated with a 1.9% increase in peak wave speed. Assuming tendon load is proportional to squared wave speed [42], this translates to a 3.8% increase in peak tendon load per degree of incline. These results agree well with a prior laboratory study that found that ankle moment increased 15.4% when treadmill incline increased from 0 to 5 degrees, which averages out to a 3.1% increase in ankle moment per degree of incline [52].

**Figure 6:**
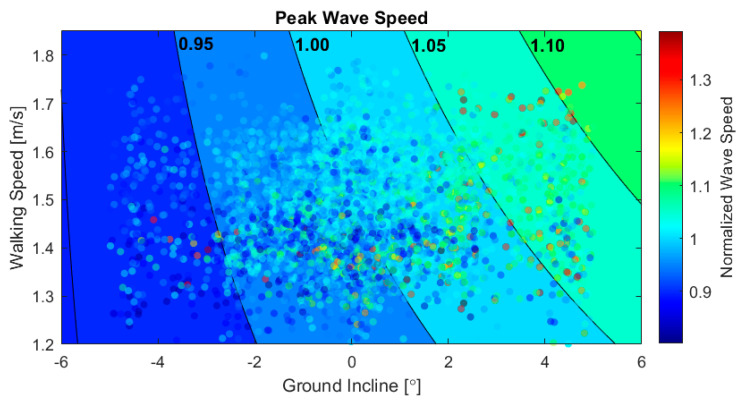
Scatter plot of peak wave speed vs. ground incline and walking speed for all 5108 strides. Data are plotted on top of contours of constant peak wave speed from a multiple linear regression with interaction.

The synchronous use of IMU and tensiometer measurements enables consideration of how both environmental and spatiotemporal aspects of walking affect tendon loading. Our multiple linear regression found a significant effect of both walking speed (*p* < 10^−6^) and walking speed-by-incline (*p* < 0.02) on peak tendon loading. The regression model was subsequentially used to generate contours of constant peak wave speed as a function of the incline and walking speed, as shown in Figure 6. This analysis revealed that peak tendon loading during push-off is diminished and relatively independent of walking speed when going down steep inclines. In contrast, walking fast up a steep incline substantially increases tendon wave speed during push-off. This regression model suggests that increasing speed from 1.25 to 1.75 m/s on −5°, 0°, and 5° ground inclines would require an increase in the tendon load of 2.2%, 8.1%, and 13.3%, respectively. The enhanced force requirement due to speed at the 0° ground incline is slightly greater than the average increases in ankle torque [53,54] and Achilles tendon loading [49] observed with controlled speed in laboratory studies. It is important to note that walking speed in this outdoors study was not controlled, such that the data could also be used to probe how environmental factors and biomechanical demands may alter individuals’ preferred speed.

**Figure 7:**
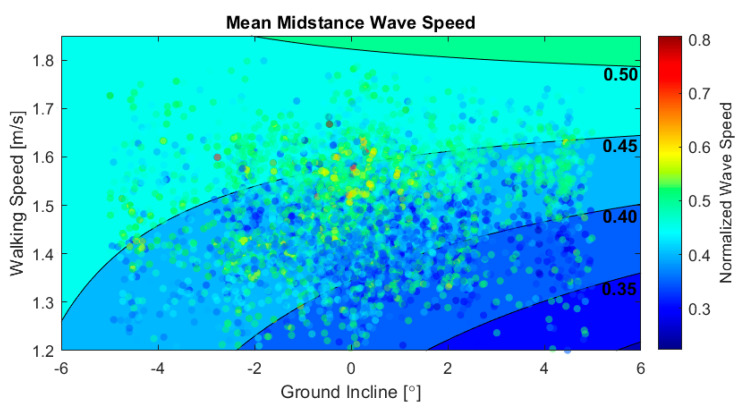
Scatter plot of the mean midstance wave speed vs. ground incline and walking speed for all 5108 strides. Data are plotted on top of contours of the constant midstance wave speed from a multiple linear regression with interaction.

We also used a coupled IMU and tensiometer analysis to investigate how individuals modulated Achilles tendon loading during the braking, i.e., midstance, phase of gait. Displayed in Figure 7, this analysis revealed that average midstance (10–30% of gait cycle) wave speed significantly decreased with terrain slope, increased with walking speed, and increased with the combined effect of these two variables (*p* < 10^−6^). The slope effect is consistent with laboratory studies of braking strategies on a negative grade [51], and likely reflects the need to push the center of pressure forward to create a rearward ground reaction force and counteract the effect of gravity. In contrast to push-off, Achilles loading during braking was also significantly affected by walking speed at all slopes. For example, the regression model suggests that increasing speed from 1.25 to 1.75 m/s on −5°, 0°, and 5° ground inclines would require an increase in tendon load of 21.6%, 62.6%, and 128%, respectively. Increasing the walking speed from 1.25 to 1.75 m/s on average induced a 29.6% increase in wave speed, and hence an estimated 67.9% increase in Achilles tendon loading in the midstance.

**Figure 8:**
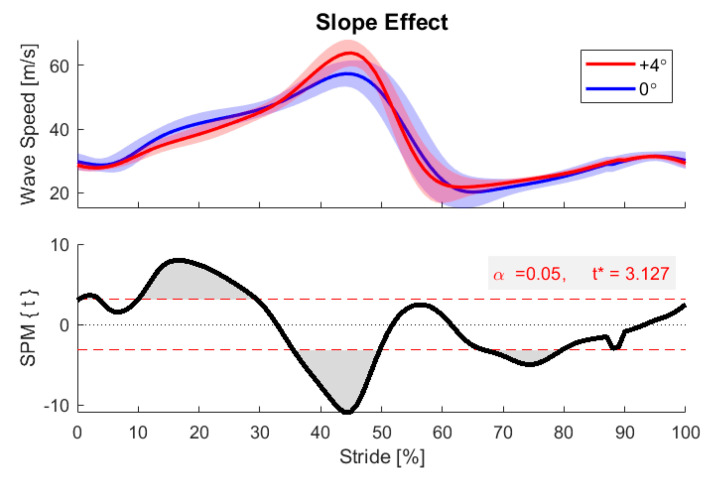
Statistical parametric mapping (SPM) between level walking (0° incline bin) and steep inclined walking (+4° bin) for one example participant. SPM revealed regions of significant difference in the two conditions (gray regions). For incline walking, meaningful differences were detected in early stance (around 20% stride) and at the push-off peak (45% stride).

An inherent challenge, and opportunity, in wearables data is the tremendous amount of data that can accumulate during real-world locomotion. Our main analysis is based on a traditional scientific approach, by sorting data records into groups to account for deterministic factors and evaluating specific outcome variables with traditional statistics [55]. However, these large data sets also provide the opportunity to employ data analytics approaches. As an example, we investigated when in the gait cycle Achilles tendon loading is directly affected by the terrain slope. To do this, we binned strides by terrain incline and then employed statistical parametric mapping (SPM) to identify periods of the gait cycle when the tendon wave speed differed from that measured during the level walking strides, as demonstrated in Figure 8 [46,47,48]. SPM analysis identified periods of the gait cycle when terrain slope could explain significant variations in tendon wave speed (Figure 8), including midstance (6 of 9 participants) and push-off (9 of 9 participants). The differences identified on a participant-specific basis were decreased braking and increased push-off when walking up inclines. As SPM can be used to compare any two loading profiles, this method could be used to address topics such as clinical differences in gait on different terrain or bilateral symmetry in recovering athletes. SPM is particularly well-suited for exploratory analyses where the effects are not predicted a priori.

**Figure 9:**
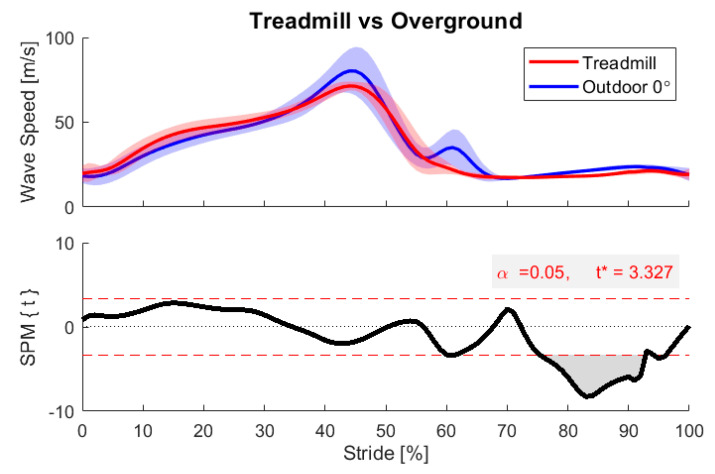
Statistical parametric mapping between in-lab and outdoor walking (0° incline bin) for one participant. SPM revealed a region of significant difference between these conditions (gray region), here in the swing phase. Greater variability was observed in the outdoor condition.

An important consideration when using wearables is whether behavior observed in the real world is distinct from that measured in laboratory conditions. We investigated this question by using SPM to compare Achilles tendon wave speeds between laboratory treadmill walking and outdoor walking strides in the 0° incline bin. This analysis revealed a tendency toward decreased Achilles tendon loading during braking and increased wave speed during push-off when walking outdoors, demonstrated by Figure 9. We also tended to observe greater Achilles tendon wave speed in the swing. Some of these differences are likely due to variations in walking speed, as our test participants elected to walk substantially faster outdoors (1.46 ± 0.16 m/s) than they had warmed up on the treadmill (1.25 m/s). However, we also observed greater variability in the outdoor conditions, which likely reflects the many unregulated factors present in the outdoor condition, such as varying terrain, non-constant speed, visibility, terrain imperfections, and other environmental distractions. Similarly, prior wearable studies have also reported greater short- and long-term variability in gait metrics, such as stride time, stride length, and cadence, when comparing treadmill and overground walking [56,57].

There are some limitations to consider when interpreting the results of this study. First, we did not include a sensor to directly detect heel strike. We also did not include a refined synchronization protocol to ensure precise alignment of tensiometry and IMU data. Instead, we aligned peak loading values to 45% of the stride, as was done in a prior study [49]. Synchronization will become important for future analysis of combined tensiometry and kinematic data, such as analyzing the precise timing of load variations and computing musculotendon power and work loops [50,58]. Second, we did not include a participant-specific calibration protocol to characterize absolute Achilles tendon loading from the measured wave speeds [49]. The measured wave speed can be affected by inherent variability in participants as well as uncertainty in the effective contact point under accelerometers of finite width. Therefore, for this study, we focused on relative differences between conditions in which a nominal condition (e.g., flat overground walking) served as the norm. We have introduced objective calibration approaches for shear wave tensiometry elsewhere, for cases when there is a need for absolute tendon load data [49,59], for example, to estimate tibial bone loading [36,37]. Finally, out of 12 participants tested, 3 were discarded due to faulty data. We also collected data from the stair segments present on the outdoor course but excluded it due to both faulty data and behavioral variability, such as different stair-descent strategies. Faulty data most often resulted from inadequate contact between the tensiometer and tendon. Reliable mounting of the shear wave tensiometer over a tendon requires training and practice, much as EMG electrodes do, and best practices remain under development to reduce the occurrence of faulty data.

Specific improvements are planned for the wearable tensiometer system to enhance its function, convenience, and utility. First, we are working to combine the drive electronics and the sensing electronics into a single compact unit for improved wearability in more dynamic movements. To enable more complete biomechanical analysis than tendon wave speed alone, we are working on full synchronization with wearable kinematic measurements as well as geographical localization through synchronous GPS and pedestrian dead-reckoning [55]. We plan to develop mounting sleeves to reliably secure the tensiometer over the Achilles tendon, reducing the dependence on expert mounting. In the longer term, we will develop such mountings for other tendons, such as the patellar and hamstring tendons [42]. We are also developing real-time analysis and display technology to enable rapid feedback to users. We anticipate that these improvements to the tensiometer device, mounting techniques, and integration with other sensors will make wearable tendon tensiometry a simple and common technology for biomechanical assessment.

Like all instrumentation, tendon tensiometry has characteristic strengths and weaknesses that affect its utility in different scenarios. As a means of estimating fluctuations in tendon load without the need for other sensors, tendon tensiometry is ideal for use cases where tendon load is the quantity directly under study. For example, studies of tendinopathy, tendon laxity, or muscle spasticity could use tensiometry as a standalone measurement. On the other hand, accelerometer-based tensiometry can only be applied to certain muscle-tendon units that have a superficial tendon accessible through a thin layer of skin, such as the Achilles and patellar tendons. Muscles with only deep tendons, extended aponeuroses, or thick layers of superficial tissue are not good candidates for this type of measurement. As a technology that relies on close-fitting contact with the body, a tendon tensiometer is well-suited for incorporation into clothing, such as exercise clothing, work clothing for heavy labor, or exosuits. Such methods of anchoring to the body are a natural extension of the straps used in this study and could improve attachment stability. Furthermore, because tensiometry is based on the timing of a wave, it is robust to moderate variations in signal amplitude that would confound other measurements like electromyography. Thus, it holds significant potential for making stable and comparable load measurements during long experiments and even across multiple days, which could be beneficial for studies of fatigue, training, rehabilitation, and other repeated-measures scenarios. On the other hand, it is sensitive to sensor placement, so current versions of the sensor must be tightened to ensure tendon contact and prevent relative motion. Improvements in the human factors aspects of the interface will be important to mitigate discomfort and enable longer duration collections. Finally, the relatively high wave speed sampling rate (100 Hz in this study) of tendon tensiometry allows it to capture detailed loading profiles at a bandwidth high enough to cover most human-initiated movements. However, this sampling rate may not be high enough to measure certain externally induced loads, such as impacts or vibrations induced by power tools or heavy machinery. Such vibrations may also interfere with the shear wave-tracking process that underpins the wave speed measurement. Thus, certain workplace and ergonomic applications may be challenging for measurement using this technology.

The development of a wireless tensiometer device creates many new potential applications in orthopedics, athletics, rehabilitation, basic biomechanical science, robotics, and ergonomics. Because of the strong relationship between the pathology and metrics of movement kinetics [60], the wearable shear wave tensiometry system could serve as an effective tool for evaluating performance, rehabilitation recovery, and overuse injury risk. For example, physical therapists and athletic trainers could someday use this device to assess symmetry and recovery to determine an injured athlete’s ability to return to sport following orthopedic surgery or other treatment. Coaches could use it to evaluate tendon loading at different levels of athletic performance or throughout long-term training. Athletes and therapists could use a real-time display for biofeedback during exercises to optimize training treatment. It may even be possible to assess workplace safety by gathering data on soft tissue loads during repetitive tasks to prevent overuse injuries, to the musculoskeletal system overall or through cumulative damage to the tendon itself [61]. Such use could improve the ergonomics of human–robot collaboration [62,63,64]. For example, repetitive tasks could be made safer and non-exhaustive by incorporating feedback regarding the exertion and fatigue level of the worker in real time, or measurements of load on a human could be used to prepare a robot to smoothly accept hand-offs of items of varying weight. Finally, the field of musculoskeletal biomechanical modeling may be greatly enhanced by the addition of tendon tensiometry data, which offers the first data on muscle-tendon loads internal to the body with which to validate such models and constrain their results. This unique and convenient new technology will enable a wide variety of new research studies and real-world applications.

## 4. Conclusions

This study introduced the use of wearable shear wave tensiometers to characterize tendon kinetics simply and noninvasively during unconstrained locomotion. From thousands of outdoor strides coupling tendon load measurements with stride-by-stride terrain characteristics, we observed characteristic changes in Achilles tendon loading that could be attributed to external factors (ground incline) and spatiotemporal aspects of walking (gait speed). Increasing uphill slope leads to decreasing tension at mid-stance and increasing tension at the push-off peak. Faster walking leads to increasing Achilles tension at both times and interacts with the effects of incline. Outdoor walking is more variable than treadmill walking, highlighting the importance of real-world data collections to understanding movement. Planned improvements, such as miniaturization and synchronization with other sensors, promise to enable new analyses of muscle-tendon loading in a variety of contexts, such as health and rehabilitation, sports performance, and ergonomics.

## Appendix A

**Table A1:** Participant characteristics for the analyzed healthy young adults.

Participant	Gender	Age (years)	Height (m)	Weight (kg)	Strides Analyzed
1	M	24	1.90	85.0	580
2	M	24	1.96	83.9	588
3	F	26	1.63	56.7	624
4	M	25	1.83	88.5	505
5	F	19	1.83	68.0	545
6	M	23	1.88	74.8	505
7	F	23	1.65	70.3	534
8	M	24	1.70	70.0	614
9	M	23	1.85	90.7	613
Mean	3F/6M	23.44 ± 1.94	1.80 ± 0.12	76.4 ± 11.3	567.6 ± 46.7

**Table A2:** Calculated gait and wave speed characteristics for analyzed healthy young adults.

Participant	Stride Length (m)	Ground Incline ^1^ (degrees)	Walking Speed (m/s)	Minimum Wave Speed ^2^ (m/s)	Peak Wave Speed ^2^ (m/s)
1	1.60 ± 0.079	0.11 ± 1.93	1.46 ± 0.10	15.13 ± 0.75	59.08 ± 6.82
2	1.68 ± 0.085	−0.08 ± 1.96	1.51 ± 0.17	14.78 ± 0.95	59.39 ± 1.90
3	1.46 ± 0.139	0.17 ± 1.96	1.42 ± 0.15	17.82 ± 1.92	78.68 ± 5.63
4	1.54 ± 0.059	0.22 ± 1.95	1.34 ± 0.19	15.31 ± 0.78	82.36 ± 11.91
5	1.52 ± 0.114	−0.02 ± 1.96	1.53 ± 0.20	13.35 ± 0.97	62.16 ± 2.43
6	1.69 ± 0.087	0.15 ± 1.97	1.57 ± 0.13	15.82 ± 0.65	58.06 ± 1.85
7	1.53 ± 0.136	0.23 ± 1.92	1.56 ± 0.10	13.33 ± 0.56	53.16 ± 2.08
8	1.45 ± 0.115	0.21 ± 1.94	1.42 ± 0.12	17.98 ± 3.70	59.11 ± 3.08
9	1.53 ± 0.102	0.24 ± 1.84	1.37 ± 0.10	15.92 ± 0.43	79.99 ± 9.41
Mean	1.55 ± 0.134	0.14 ± 1.94	1.46 ± 0.16	15.58 ± 2.23	66.13 ± 12.23

^1^ Ground Incline is the mean incline of strides in the zero-degree binned condition for each participant, which captured all individual strides between −1 and +1 degrees. ^2^ Minimum and peak wave speeds were obtained from strides in the zero-degree binned condition to allow for comparison to previous studies involving level treadmill walking.

## Appendix B

**Table A3:** Individual linear regressions ^1^ of normalized peak wave speed with respect to ground incline (degrees).

Participant	Coefficient	Intercept	*p*-Value	R^2^
1	0.0443	0.9948	1.17 × 10^−54^	0.3428
2	0.0053	1.0004	1.58 × 10^−13^	0.0888
3	0.0276	0.9954	8.70 × 10^−58^	0.3384
4	0.0184	0.9959	7.17 × 10^−8^	0.0561
5	0.0176	1.0003	4.18 × 10^−82^	0.4929
6	0.0137	0.9979	1.30 × 10^−60^	0.4154
7	0.0149	0.9966	9.00 × 10^−46^	0.3158
8	0.0117	0.9975	7.11 × 10^−21^	0.1338
9	0.0182	0.9956	1.06 × 10^−10^	0.0660
Overall	0.0191	0.9974	2.96 × 10^−184^	0.1514

^1^ Linear regressions were performed on individual participant data, normalized to their mean peak wave speed at the zero-degree incline binned condition.

## References

[1] Chambers R., Gabbett T.J., Cole M.H., Beard A. (2015). The Use of Wearable Microsensors to Quantify Sport-Specific Movements. Sports Med..

[2] Li R.T., Kling S.R., Salata M.J., Cupp S.A., Sheehan J., Voos J.E. (2016). Wearable Performance Devices in Sports Medicine. Sports Health.

[3] Peppoloni L., Filippeschi A., Ruffaldi E., Avizzano C.A. (2014). (WMSDs Issue) A Novel Wearable System for the Online Assessment of Risk for Biomechanical Load in Repetitive Efforts. Int. J. Ind. Ergon..

[4] Alberto R., Draicchio F., Varrecchia T., Silvetti A., Iavicoli S. (2018). Wearable Monitoring Devices for Biomechanical Risk Assessment at Work: Current Status and Future Challenges—A Systematic Review. Int. J. Environ. Res. Public Health.

[5] Conforti I., Mileti I., Del Prete Z., Palermo E. Assessing Ergonomics and Biomechanical Risk in Manual Handling of Loads through a Wearable System. Proceedings of the 2019 IEEE International Workshop on Metrology for Industry 4.0 and IoT, MetroInd 4.0 and IoT.

[6] Kwon J., Park J.H., Ku S., Jeong Y.H., Paik N.J., Park Y.L. (2019). A Soft Wearable Robotic Ankle-Foot-Orthosis for Post-Stroke Patients. IEEE Robot. Autom. Lett..

[7] Iosa M., De Sanctis M., Summa A., Bergamini E., Morelli D., Vannozzi G. (2018). Usefulness of Magnetoinertial Wearable Devices in Neurorehabilitation of Children with Cerebral Palsy. Appl. Bionics Biomech..

[8] Havens K.L., Cohen S.C., Pratt K.A., Sigward S.M. (2018). Accelerations from Wearable Accelerometers Reflect Knee Loading during Running after Anterior Cruciate Ligament Reconstruction. Clin. Biomech..

[9] Hullfish T.J., Qu F., Stoeckl B.D., Gebhard P.M., Mauck R.L., Baxter J.R. (2019). Measuring Clinically Relevant Knee Motion with a Self-Calibrated Wearable Sensor. J. Biomech..

[10] Camomilla V., Bergamini E., Fantozzi S., Vannozzi G. (2018). Trends Supporting the In-Field Use of Wearable Inertial Sensors for Sport Performance Evaluation: A Systematic Review. Sensors.

[11] Adesida Y., Papi E., McGregor A.H. (2019). Exploring the Role of Wearable Technology in Sport Kinematics and Kinetics: A Systematic Review. Sensors.

[12] Johansson D., Malmgren K., Alt Murphy M. (2018). Wearable Sensors for Clinical Applications in Epilepsy, Parkinson’s Disease, and Stroke: A Mixed-Methods Systematic Review. J. Neurol..

[13] Thorp J.E., Adamczyk P.G., Ploeg H.-L., Pickett K.A. (2018). Monitoring Motor Symptoms During Activities of Daily Living in Individuals With Parkinson’s Disease. Front. Neurol..

[14] Dadashi F., Mariani B., Rochat S., Büla C.J., Santos-Eggimann B., Aminian K. (2013). Gait and Foot Clearance Parameters Obtained Using Shoe-Worn Inertial Sensors in a Large-Population Sample of Older Adults. Sensors.

[15] Tunca C., Pehlivan N., Ak N., Arnrich B., Salur G., Ersoy C. (2017). Inertial Sensor-Based Robust Gait Analysis in Non-Hospital Settings for Neurological Disorders. Sensors.

[16] Rebula J.R., Ojeda L.V., Adamczyk P.G., Kuo A.D. (2013). Measurement of Foot Placement and Its Variability with Inertial Sensors. Gait Posture.

[17] Ojeda L.V., Adamczyk P.G., Rebula J.R., Nyquist L.V., Strasburg D.M., Alexander N.B. (2019). Reconstruction of Body Motion during Self-Reported Losses of Balance in Community-Dwelling Older Adults. Med. Eng. Phys..

[18] Ueberschär O., Fleckenstein D., Warschun F., Kränzler S., Walter N., Hoppe M.W. (2019). Measuring Biomechanical Loads and Asymmetries in Junior Elite Long-Distance Runners through Triaxial Inertial Sensors. Sport. Orthop. Traumatol..

[19] Sheerin K.R., Reid D., Besier T.F. (2019). The Measurement of Tibial Acceleration in Runners—A Review of the Factors That Can Affect Tibial Acceleration during Running and Evidence-Based Guidelines for Its Use. Gait Posture.

[20] Howell A.M., Kobayashi T., Hayes H.A., Foreman K.B., Bamberg S.J.M. (2013). Kinetic Gait Analysis Using a Low-Cost Insole. IEEE Trans. Biomed. Eng..

[21] Bonnet V., Mazza C., Fraisse P., Cappozzo A. (2013). Real-time Estimate of Body Kinematics During a Planar Squat Task Using a Single Inertial Measurement Unit. IEEE Trans. Biomed. Eng..

[22] Latella C., Kuppuswamy N., Romano F., Traversaro S., Nori F. (2016). Whole-Body Human Inverse Dynamics with Distributed Micro-Accelerometers, Gyros and Force Sensing. Sensors.

[23] Disselhorst-Klug C., Schmitz-Rode T., Rau G. (2009). Surface electromyography and muscle force: Limits in sEMG–force relationship and new approaches for applications. Clin. Biomech..

[24] Ginn K., Eastburn G., Lee M. (1993). Evaluation of a buckle force transducer for measuring tissue tension. Aust. J. Physiother..

[25] Erdemir A., Hamel A.J., Piazza S.J., Sharkey N.A. (2003). Fiberoptic measurement of tendon forces is influenced by skin movement artifact. J. Biomech..

[26] Komi P.V. (1990). Relevance of in vivo force measurements to human biomechanics. J. Biomech..

[27] Fukashiro S., Komi P., Järvinen M., Miyashita M. (1993). Comparison between the directly measured achilles tendon force and the tendon force calculated from the ankle joint moment during vertical jumps. Clin. Biomech..

[28] Fukashiro S., Komi P.V., Järvinen M., Miyashita M. (1995). In vivo achilles tendon loading’ during jumping in humans. Graefe’s Arch. Clin. Exp. Ophthalmol..

[29] Finni T., Komi P.V., Lukkariniemi J. (1998). Achilles tendon loading during walking: Application of a novel optic fiber technique. Graefe’s Arch. Clin. Exp. Ophthalmol..

[30] Ancillao A., Tedesco S., Barton J., O’Flynn B. (2018). Indirect Measurement of Ground Reaction Forces and Moments by Means of Wearable Inertial Sensors: A Systematic Review. Sensors.

[31] Karatsidis A., Bellusci G., Schepers H.M., De Zee M., Andersen M.S., Veltink P. (2016). Estimation of Ground Reaction Forces and Moments During Gait Using Only Inertial Motion Capture. Sensors.

[32] Schepers H.M., Koopman H.F.J.M., Veltink P. (2007). Ambulatory Assessment of Ankle and Foot Dynamics. IEEE Trans. Biomed. Eng..

[33] Hurkmans H.L., Bussmann J., Benda E., Verhaar J.A.N., Stam H. (2006). Accuracy and repeatability of the Pedar Mobile system in long-term vertical force measurements. Gait Posture.

[34] Saggin B., Scaccabarozzi D., Tarabini M. (2013). Metrological Performances of a Plantar Pressure Measurement System. IEEE Trans. Instrum. Meas..

[35] Fong D.T., Chan Y.-Y., Hong Y., Yung P.S., Fung K.-Y., Chan K.-M. (2008). Estimating the complete ground reaction forces with pressure insoles in walking. J. Biomech..

[36] Matijevich E.S., Branscombe L.M., Scott L.R., Zelik K.E. (2019). Ground reaction force metrics are not strongly correlated with tibial bone load when running across speeds and slopes: Implications for science, sport and wearable tech. PLoS ONE.

[37] Meardon S.A., Willson J.D., Gries S.R., Kernozek T.W., Derrick T.R. (2015). Bone stress in runners with tibial stress fracture. Clin. Biomech..

[38] Vergari C., Ravary-Plumioën B., Evrard D., Laugier P., Mitton D., Pourcelot P., Crevier-Denoix N. (2012). Axial speed of sound is related to tendon’s nonlinear elasticity. J. Biomech..

[39] Bercoff J., Tanter M., Fink M. (2004). Supersonic shear imaging: A new technique for soft tissue elasticity mapping. IEEE Trans. Ultrason. Ferroelectr. Freq. Control..

[40] Hug F., Tucker K., Gennisson J.-L., Tanter M., Nordez A. (2015). Elastography for Muscle Biomechanics. Exerc. Sport Sci. Rev..

[41] DeWall R.J., Slane L.C., Lee K.S., Thelen D.G. (2014). Spatial variations in Achilles tendon shear wave speed. J. Biomech..

[42] Martin J.A., Brandon S.C.E., Keuler E.M., Hermus J.R., Ehlers A.C., Segalman D.J., Allen M.S., Thelen D.G. (2018). Gauging force by tapping tendons. Nat. Commun..

[43] Martin J.A., Biedrzycki A.H., Lee K.S., DeWall R.J., Brounts S.H., Murphy W.L., Markel M.D., Thelen D.G. (2015). In Vivo Measures of Shear Wave Speed as a Predictor of Tendon Elasticity and Strength. Ultrasound Med. Boil..

[44] Bolus N., Jeong H.-K., Blaho B.M., Safaei M., Young A., Inan O. (2020). Fit to Burst: Toward Noninvasive Estimation of Achilles Tendon Load Using Burst Vibrations. IEEE Trans. Biomed. Eng..

[45] Céspedes I., Huang Y., Ophir J., Spratt S. (1995). Methods for Estimation of Subsample Time Delays of Digitized Echo Signals. Ultrason. Imaging.

[46] Pataky T.C., Robinson M.A., Vanrenterghem J. (2016). Region-of-interest analyses of one-dimensional biomechanical trajectories: Bridging 0D and 1D theory, augmenting statistical power. PeerJ.

[47] Pataky T.C. (2012). One-dimensional statistical parametric mapping in Python. Comput. Methods Biomech. Biomed. Eng..

[48] Pataky T.C. (2019). One-Dimensional Statistical Parametric Mapping in MATLAB.

[49] Keuler E.M., Loegering I.F., Martin J.A., Roth J.D., Thelen D.G. (2019). Shear Wave Predictions of Achilles Tendon Loading during Human Walking. Sci. Rep..

[50] Lichtwark G.A., Wilson A.M. (2006). Interactions between the human gastrocnemius muscle and the Achilles tendon during incline, level and decline locomotion. J. Exp. Boil..

[51] Lay A.N., Hass C.J., Gregor R.J. (2006). The effects of sloped surfaces on locomotion: A kinematic and kinetic analysis. J. Biomech..

[52] Silder A., Besier T.F., Delp S.L. (2012). Predicting the metabolic cost of incline walking from muscle activity and walking mechanics. J. Biomech..

[53] Stoquart G., Detrembleur C., Lejeune T. (2008). Effect of speed on kinematic, kinetic, electromyographic and energetic reference values during treadmill walking. Neurophysiol. Clin. Neurophysiol..

[54] Goldberg S.R., Stanhope S.J. (2013). Sensitivity of joint moments to changes in walking speed and body-weight-support are interdependent and vary across joints. J. Biomech..

[55] Wang W., Adamczyk P.G. (2019). Analyzing Gait in the Real World Using Wearable Movement Sensors and Frequently Repeated Movement Paths. Sensors.

[56] Hollman J.H., Watkins M.K., Imhoff A.C., Braun C.E., Akervik K.A., Ness D.K. (2016). A comparison of variability in spatiotemporal gait parameters between treadmill and overground walking conditions. Gait Posture.

[57] Tamburini P., Storm F.A., Buckley C., Bisi M.C., Stagni R., Mazza C. (2018). Moving from laboratory to real life conditions: Influence on the assessment of variability and stability of gait. Gait Posture.

[58] Komisar V., Novak A.C., Haycock B. (2017). A novel method for synchronizing motion capture with other data sources for millisecond-level precision. Gait Posture.

[59] Ebrahimi A., Martin J.A., Schmitz D.G., Thelen D.G. (2020). Shear Wave Tensiometry Reveals an Age-Related Deficit in Triceps Surae Work at Slow and Fast Walking Speeds. Front. Sports Act. Living.

[60] Schmitt L.C., Paterno M.V., Ford K.R., Myer G.D., Hewett T.E. (2015). Strength Asymmetry and Landing Mechanics at Return to Sport after Anterior Cruciate Ligament Reconstruction. Med. Sci. Sports Exerc..

[61] Firminger C.R., Asmussen M.J., Cigoja S., Fletcher J.R., Nigg B.M., Edwards W.B. (2020). Cumulative Metrics of Tendon Load and Damage Vary Discordantly with Running Speed. Med. Sci. Sports Exerc..

[62] Roy S., Edan Y. (2018). Investigating Joint-Action in Short-Cycle Repetitive Handover Tasks: The Role of Giver Versus Receiver and its Implications for Human-Robot Collaborative System Design. Int. J. Soc. Robot..

[63] Kim W., Lee J., Peternel L., Tsagarakis N., Ajoudani A. (2017). Anticipatory Robot Assistance for the Prevention of Human Static Joint Overloading in Human–Robot Collaboration. IEEE Robot. Autom. Lett..

[64] Someshwar R., Meyer J., Edan Y. (2012). A Timing Control Model for H-R Synchronization. IFAC Proc. Vol..

